# Conservative Management of Mesenteric Panniculitis in a Remote Island

**DOI:** 10.1155/2023/3335738

**Published:** 2023-04-21

**Authors:** Paschalis Gavriilidis, Nicola de' Angelis

**Affiliations:** ^1^Department of Surgery, Saint Helena General Hospital, Jamestown, STHL 1ZZ, Saint Helena, UK; ^2^Colorectal and Digestive Surgery Unit at Hospital Beaujon, 100 Bd du Général Leclerc, Clichy 92110, France

## Abstract

**Introduction:**

Mesenteric panniculitis (MP) includes a spectrum of nonspecific fibroinflammatory disorders of unknown aetiology that affects mainly the root of the mesentery. *Case Report*. A 68-year-old man is incidentally diagnosed with MP during follow-up investigation for a fusiform coeliac artery aneurysm. Four years since the diagnosis, he is completely asymptomatic. After discussing with him and presenting the current evidence, he decided not to proceed with biopsy because the finding was incidental and he is asymptomatic. Moreover, tumour markers were within the normal range. He has been scheduled for annual follow-ups with computerized tomography (CT) scans and tumour markers.

**Conclusions:**

MP is a rare chronic fibroinflammatory disease with contradictory evidence regarding its definition and management. Watchful follow-ups with CT scan and tumour markers are recommended for asymptomatic patients.

## 1. Introduction

Mesenteric panniculitis (MP) was first described in 1924 by Jura [1]. He used the term “retractile mesenteritis.” Consequently, in 1955, Crane et al. presented five cases and described the fibroinflammatory disorder at the root of the mesentery with the term “mesenteric lipodystrophy” [2]. Finally, in 1960, Ogden et al. introduced the term “MP” [3].

Usually, MP is detected incidentally during follow-up investigations [4]. The reported prevalence rate varies from 0.6% to 2.5% [5, 6]. The prevalent symptoms in symptomatic patients are vague abdominal pain and fullness in the central abdomen and upper left quadrant, nausea, weight loss, and change of bowel habits; they may be progressive or intermittent [7].

Histological classification is based on the following three pathological characteristics: chronic nonspecific inflammation, fat necrosis, and fibrosis [7, 8].

For many decades, the varied terminology caused considerable confusion. However, the classification based on the above three pathological characteristics helped to classify the condition as a single disease with two pathological subgroups. In particular, if chronic inflammation and fat fibrosis prevail over fibrosis, the disease is defined as MP, and when fibrosis and retraction are the main pathological characteristics, the condition is defined as sclerosing mesenteritis (SM). The term “retractile mesenteritis” is also used interchangeably with SM [8].

Mesenteric ischaemia provoked by the compression of the encapsulated mass at the root of the mesentery and autoimmune response to unknown sources considered so far the causal mechanisms of the disease [9, 10].

The associated rate of MP with malignancy varies from 38% to 50% of cases [11, 12].

Corticosteroids, thiopurines, colchicine, thalidomide, and tamoxifen have been used for conservative management; surgical intervention is limited only for complications, and any attempt for complete resection is considered technically not feasible and of no profit [13, 14].

The main aim of this study was to present a 68-year-old man diagnosed incidentally with MP four years ago and to review the literature referring to the definition, diagnosis, and management of the disease.

## 2. Case Presentation

**Figure 1:**
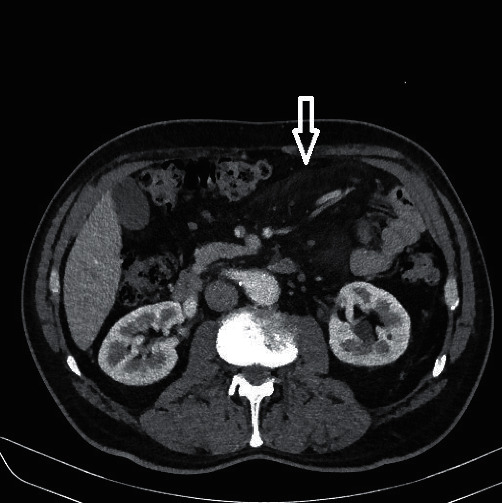
(Arrow) well-circumscribed misting of the mesentery (centrally and left-sided mass) with subcentimetre mesenteric lymph nodes consistent with MP.

A 68-year-old man, five years ago, underwent computerized tomography (CT) scan for nonspecific abdominal pain and diagnosed with fusiform coeliac artery aneurysm approximately 15 mm from the origin measuring 20 mm × 17 mm with CT scan. Since then, he did not complain for any recurrence of the abdominal pain. Two years later, during surveillance CT scan, he has been diagnosed with findings suggestive of MP. In the second surveillance CT scan four years later, the MP is described as “well-circumscribed misting of the mesentery with subcentimetre mesenteric lymph nodes consistent with mesenteric panniculitis. This is unchanged in appearance with no new abdominal findings. Specifically, no size was significant abdominal adenopathy compared with previous CT” (Figure 1). He has been further investigated with carbohydrate antigen (CA 19-9), carcinoembryonic antigen (CEA), and colonoscopy to rule out any underlying malignancy. Nothing abnormal was detected from the results of the tumour markers and colonoscopy. The Patient is referred by his General Practitioner with the question whether biopsy is needed for further confirmation of the radiological diagnosis. The current evidence is presented to the patient, and he decided not to proceed with biopsy for an incidentally discovered asymptomatic finding. Patient is scheduled for annual follow-ups with CT scan for his comorbidities, and he will undergo CEA and CA19-9 investigations to rule out the occurrence of a new malignancy. Surveillance colonoscopy has been scheduled for every three years, although there is a lack of guidelines about that.

## 3. Discussion

MP is a spectrum disease, and there are ongoing debates on many topics of natural history of the disease.

Regarding the age of onset, there is consent between the studies so far that MP occurs usually between the age of 50 and 70 years [5, 15–17].

However, there is a discrepancy regarding the gender distribution; three studies reported that men to women ratio is 3 : 1. However, Daskalogiannaki et al. reported a higher prevalence of women 65% of the patients [5, 16, 18].

The topic that triggered an interesting debate was the relationship and association between MP and underlying malignancy. Furthermore, many researchers by studying the natural history of MP in relation with malignancy status tried to answer the question whether MP is a paraneoplastic phenomenon or an epiphenomenon of the coexisting malignant disease.

In particular, the prevalence rate of malignancies diagnosed with coexisting MP is lymphoma 28%, melanoma 18%, colorectal cancer 15%, and prostate cancer 13%. Moreover, it has been reported that the overall association rate of MP with coexisting neoplasia is 56%. The above findings made the authors to conclude that MP is a paraneoplastic disease [18]. However, Buchwald et al. demonstrated that MP is an epiphenomenon rather than a paraneoplastic phenomenon of underlying malignancy; the above conclusion was based on the observation that MP did not regress when the coexisted malignancy responded to treatment. Moreover, he did not detect any significant regression rate of MP between the cohorts of patients with and without an underlying malignancy [19].

In order to avoid the risk of overdiagnosis or underdiagnosis, CT scan diagnostic criteria were formulated.

In 2011, Coulier summarised the hallmark characteristics for the CT scan diagnosis of MP by the following five criteria: (1) detection of a well-defined “mass effect” on adjacent structures and organs, (2) this pressure is due to a mass with characteristic heterogenous higher attenuation compared with the surrounding retroperitoneal and mesocolonic fat, (3) and contains numerous little hazy and hypodense soft tissue nodes, (4) which in most of the cases may surrounded by characteristic “halo sign”, and (5) and characteristic pseudocapsule may envelope the whole mass [15].

Special attention should be given to the numerous diseases that are included in the differential diagnosis. The most prevalent are lymphoma, well-differentiated liposarcoma, peritoneal carcinomatosis, retroperitoneal fibrosis, carcinoid tumour, mesenteric fibromatosis, desmoid tumour, and mesenteric oedema. In addition, hyper-attenuated mesenteric fat may present portal hypertension, mesenteric oedema, mesenteric trauma, or even neoplastic infiltration of the mesenteric root. [15, 16]. In case of diagnostic dilemma, Positron Emission tomography (PET)/CT is a useful tool to differentiate between malignancy and MP [15].

This study presents a patient with CT scan diagnosis of MP. Four years since the diagnosis, he is asymptomatic, and the tumour markers and surveillance colonoscopy nothing abnormal were detected. Therefore, based on the current evidence, the indicated management consists of surveillance CT scan and tumour markers to rule out an occurrence of new malignancy. Of note, symptomatic patients are candidates for treatment with steroids and immunosuppressants, and surgery is limited only to complications [11–14].

## 4. Conclusions

Every patient incidentally diagnosed with MP should undergo detailed investigation to rule out an underlying malignancy. Tumour markers of the most prevalent associated malignancies are helpful, and in case of diagnostic dilemma, PET/CT can help to exclude malignancy. Follow-up investigation should include CT scan and tumour markers. Surgical intervention is indicated only for complications.

## Data Availability

The authors declare that data supporting the findings of this study are available within the article.

## Additional Points

*Learning Points.* The learning points are as follows. (1)Chronic nonspecific inflammation, fat necrosis, and fibrosis are the hallmark histological characteristics of the condition. (2) In MP, chronic inflammation and fat necrosis prevail over fibrosis. (3) Fibrosis is the main histological characteristic of SM. (4) Follow-up of asymptomatic patients consists of CT scan and tumour markers to rule out associated or new malignancy. (5) First-line treatment for symptomatic patients is steroids and immunosuppressants. (6) Surgery is limited only to complications.

## References

[1] Jura V. (1924). Sulla mesenterite retractile e sclerosante. *Policlinico*.

[2] Crane J. T., Aguilar M. J., Grimes O. F. (1955). Isolated lipodystrophy, a form of mesenteric tumor. *American Journal of Surgery*.

[3] Ogden W. W., Bradburn D. M., Rives J. D. (1960). Panniculitis of the mesentery. *Annals of Surgery*.

[4] Khachaturian T., Hughes J. (1988). Mesenteric panniculitis. *Western Journal of Medicine*.

[5] Daskalogiannaki M., Voloudaki A., Prassopoulos P. (2000). CT evaluation of mesenteric panniculitis. *American Journal of Roentgenology*.

[6] Van Putte-Katier N., van Bommel E. F., Eglersma O. E., Hendriksz T. R. (2014). Mesenteric panniculitis: prevalence, clinicoradiological presentation and 5-year follow-up. *The British Journal of Radiology*.

[7] Emory T. S., Monihan J. M., Carr N. J., Sobin L. H. (1997). Sclerosing mesenteritis, mesenteric panniculitis and mesenteric lipodystrophy: a single entity?. *The American Journal of Surgical Pathology*.

[8] Vettoretto N., Diana D. R., Poiatti R., Matteucci A., Chioda C., Giovanetti M. (2007). Occasional finding of mesenteric lipodystrophy during laparoscopy: a difficult diagnosis. *World Journal of Gastroenterology*.

[9] Issa I., Baydoun H. (2009). Mesenteric panniculitis: various presentations and treatment regimens. *World Journal of Gastroenterology*.

[10] Wilkes A., Griffin N., Dixon L., Dobbs B., Frizelle F. A. (2012). Mesenteric panniculitis. *Diseases of the Colon and Rectum*.

[11] Cross A. J., McCormick J., Griffin N., Dixon L., Dobbs B., Frizelle F. (2016). Malignancy and mesenteric panniculitis. *Colorectal Disease*.

[12] Akram S., Pardi D. S., Schaffner J. A., Smyrk T. C. (2007). Sclerosing mesenteritis: clinical features, treatment and outcome in ninety-two patients. *Clinical Gastroenterology and Hepatology*.

[13] Nyberg L., Björk J., Björkdahl P., Ekberg O., Sjöberg K., Virgen L. (2017). Sclerosing mesenteritis and mesenteric panniculitis-clinical experience and radiological features. *BMC Gastroenterology*.

[14] Durst A. L., Freund H., Rosenmann E., Birnbaum D. (1977). Mesenteric panniculitis: review of the literature and presentation of cases. *Surgery*.

[15] Coulier B. (2011). Mesenteric panniculitis. Part 1: MDCT—pictorial review. *JBR-BTR*.

[16] Coulier B. (2011). Mesenteric panniculitis. Part 2: prevalence and natural course: MDCT prospective study. *JBR-BTR*.

[17] Smith Z. L., Sifuentes H., Deepak P., Ecanow D. B., Ehrenpreis E. D. (2013). Relationship between mesenteric abnormalities on computed tomography and malignancy: clinical findings and outcomes of 359 patients. *Journal of Clinical Gastroenterology*.

[18] Badet N., Sailley N., Briquez C., Paquette B., Vuitton L., Delabrousse E. (2015). Mesenteric panniculitis: still an ambiguous condition. *Diagnostic and Interventional Imaging*.

[19] Buchwald P., Diesing L., Dixon L. (2016). Cohort study of mesenteric panniculitis and its relationship to malignancy. *The British Journal of Surgery*.

